# A Critical Dose of Doxorubicin Is Required to Alter the Gene Expression Profiles in MCF-7 Cells Acquiring Multidrug Resistance

**DOI:** 10.1371/journal.pone.0116747

**Published:** 2015-01-30

**Authors:** Shang-Hsun Tsou, Tzer-Ming Chen, Hui-Ting Hsiao, Yen-Hui Chen

**Affiliations:** 1 Graduate Institute of Pharmaceutical Sciences, School of Pharmacy, College of Medicine, National Taiwan University, Taipei, Taiwan; 2 Department of Obstetrics and Gynecology, College of Medicine, National Taiwan University, Taipei, Taiwan; 3 Graduate Institute of Clinical Pharmacy, School of Pharmacy, College of Medicine, National Taiwan University, Taipei, Taiwan; Seoul National University, KOREA, REPUBLIC OF

## Abstract

Cellular mechanisms of multidrug resistance (MDR) are related to ABC transporters, apoptosis, antioxidation, drug metabolism, DNA repair and cell proliferation. It remains unclear whether the process of resistance development is programmable. We aimed to study gene expression profiling circumstances in MCF-7 during MDR development. Eleven MCF-7 sublines with incremental doxorubicin resistance were established as a valued tool to study resistance progression. MDR marker P-gp was overexpressed only in cells termed MCF-7/ADR-1024 under the selection dose approaching 1024 nM. MCF-7/ADR-1024 and authentic MCF-7/ADR shared common features in cell morphology and DNA ploidy status. MCF-7/ADR-1024 and authentic MCF-7/ADR down regulated repair genes BRCA1/2 and wild type p53, apoptosis-related gene Bcl-2 and epithelial-mesenchymal transition (EMT) epithelial marker gene E-cadherin. While detoxifying enzymes glutathione-S transferase-π and protein kinase C-α were up-regulated. The genes involving in EMT mesenchymal formation were also overexpressed, including N-cadherin, vimentin and the E-cadherin transcription reppressors Slug, Twist and ZEB1/2. PI3K/AKT inhibitor wortmannin suppressed expression of Slug, Twist and mdr1. Mutant p53 with a deletion at codons 127-133 markedly appeared in MCF-7/ADR-1024 and authentic MCF-7/ADR as well. In addition, MCF-7/ADR-1024 cells exerted CSC-like cell surface marker CD44 high/CD24 low and form mammospheres. Overall, results suggest that resistance marker P-gp arises owing to turn on/off or mutation of the genes involved in DNA repair, apoptosis, detoxifying enzymes, EMT and ABC transporters at a turning point (1.024 μM doxorubicin challenge). Behind this point, no obvious alterations were found in most tested genes. Selection for CSC-like cells under this dose may importantly attribute to propagation of the population presenting invasive properties and drug resistance. We thereby suggest two models in the induction of drug resistance. Model 1: Selection for CSC-like cells. Model 2: Mutations for gain-of resistance. Either model 1 or model 2 requires doxorubicin dose approaching 1 μM to alter gene regulation.

## Introduction

The ability of cancer cells to become simultaneously resistant to different drugs—a trait known as multidrug resistance (MDR)—remains a significant impediment to successful chemotherapy [1, 2]. The mechanisms of MDR development have been studied extensively because the MDR constitutes a major factor to the reduced efficacy of many chemotherapeutic agents. Several hypotheses have been proposed to account for the phenomenon of MDR including activation of DNA repair pathways, alteration of drug targets, decreased uptake of chemotherapeutic drugs, and most importantly, an increased active efflux of drugs mediated by transporters belonging to the ATP binding cassette (ABC transporters) superfamily of proteins [3, 4]. Elevated expression of membrane drug efflux pumps such as P-glycoprotein (P-gp, ABCB1), multidrug resistance protein 1 (MRP-1, ABCC1) and ABCG2 is a frequent cause of MDR in human cancers [5, 6]. Experimental models for MDR can be easily generated by *in vitro* selection with cytotoxic agents [7–9]. However, the mechanism of sequential development of MDR is still unclear as most experiments were designed for comparison of the wild type with the resistant type cells [10]. The increase in mdr1 gene expression is observed prior to gene amplification and P-gp increases with concurrent transcripts of the resistance-related genes, suggesting that activation of the MDR phenotype is complex [11–13].

The second way by which tumor cells can circumvent the cytotoxic action of chemotherapeutic drugs is the increased detoxification by metabolizing enzymes, antioxidation enzymes, etc. In resistant tumor cells, gene overexpression was found in drug metabolizing enzymes such as glutamate–cysteine ligase (GCL) and glutathione S-transferases (GSTs) [14, 15]. Nrf-2 is known as a major transcription factor that mediates ARE-driven transcription. Nrf-2 regulates the antioxidant response by introducing the expression of genes bearing an ARE in their regulatory regions, such as γ-GCL, and HO-1[14, 16]. Activation of the Nrf-2 pathway composes a cellular protective system that promotes cell survival under detrimental environments.

Another way of obtaining MDR is alterations in target molecules. Tumor cells can become resistant due to the enhanced repair of DNA. Alkylating agents react with DNA to form DNA-adducts, leading to DNA lesions. BRCA-1 and BRCA-2 encode proteins that are crucial for the accurate repair of DNA double strand breaks and the expression of BRCA-1/2 increases in MDR cells [17]. Changes in genes that are critical for proliferation or apoptosis can lead to the abrogation of apoptosis or cell cycle arrest. The p53 protein is an important protein in the regulation of the cell cycle and the induction of apoptosis in response to DNA damage. Deletions and mutations of p53 have been observed in several MDR tumor cell lines and cause a loss of function of p53 [8, 18–20].

There are additional hypotheses to elucidate drug resistance development including cancer stem cell (CSC) theory, cell signaling changes and mutation accumulation [21–23]. The concept of CSC-like cells is currently employed to explain the mechanism of MDR. It is generally believed that conventional chemotherapy is incapable of killing all the tumor cells. A small subpopulation of cells which are resistant to the chemotherapy and have the capability of initiating tumors may be the source of recurrence. These cells are referred as CSC-like cells [24]. Tumors are clearly histologically heterogeneous with subsets of cancer cells exhibiting distinct molecular profiles [25]. Cells with different molecular characteristics within the same tumor may undergo adaptive changes after therapy, leading to drug resistance. In epithelial cancers, these adaptive changes may involve epithelial to mesenchymal transition (EMT) and the reverse process, mesenchymal to epithelial transition. EMT can trigger conversion to a CSC-like phenotype, providing an association between EMT, CSCs and drug resistance [26]. Detection of CSC-related markers and the EMT properties will be a clue for the elevated expression of ABC transporters in cells developing drug resistance. Besides, various signaling pathways and transcription factors are known to influence the response to P-gp-mediated MDR, including the phosphatidylinositol 3-kinase (PI3K)/protein kinase B (AKT) pathway, p53, protein kinase C and other protein kinases [20, 27, 28].

Doxorubicin is an anthracycline drug that intercalates DNA and inhibits DNA replication. Applications of doxorubicin cause development of MDR and gain P-gp expression in several tumors [7, 29, 30]. We intended to understand the effect of doxorubicin treatment on the development of drug resistance in tumors and more importantly to understand how the process was sequentially affected by a set of genes. The parent MCF-7 used in this study is a human adenocarcinoma cell line that retains several characteristics of differentiated mammary epithelium such as the ability to process estrogen via estrogen receptors. Resistant cells were selected by doxorubicin at the starting concentration of 1 nM. After cells were tolerable, a double concentration of doxorubicin was applied. The process was repeatedly performed to increase cell tolerance to doxorubicin. As the doxorubicin selection dose up to 1024 nM, the cells gained resistance phenotypes similar to the authentic MCF-7/ADR cells. We employed the series of MCF-7 sublines to detect the expression profiles of genes involving in drug resistance development. Using the series of MCF-7 sublines, we can not only understand what genes involved in drug resistance development, but also find the progressively regulatory machineries in the induction of MDR. With regard to this study, we found that development of resistance was associated with mutations in repair system, the presence of CSC properties, the EMT properties, increased expression of detoxifying genes and elevated ABC transporters. Selection of CSC-like cells may be an important impact to promote propagation of the population that expresses drug resistance phenotypes.

## Materials and Methods

### Antibodies and chemicals

Doxorubicin, verapamil, calcein-AM, rhodamine 123 and MK571 were purchased from Sigma (Sigma, St. Louis, MO). The following antibodies were employed in this study. Anti-P-gp and anti-Nrf-2 (sc-722, sc-13032) were purchased from Santa Cruz Biotechnology (Santa Cruz, CA, USA). Anti-MRP1 was from Chemicon (Chemicon, Temecula, CA) and anti-p53 was from Epitomics (Epitomics, Burlingame, CA).

### Cell culture

MCF-7/ADR (ADR: Adriamycin resistance), a doxorubicin-selected P-glycoprotein overexpressed cell line, derived from the human breast cancer cell line MCF-7, was maintained in the presence of 1 μM doxorubicin. A series of MCF-7 cells with incremental resistance to doxorubicin were established by doxorubicin challenge at the starting concentration of 1 nM. After cells were tolerable, a double concentration of doxorubicin was applied. The process was repeatedly performed to increase cell tolerance to doxorubicin. The resulting 11 sublines, MCF-7 and MCF-7/ADR were cultured in DMEM (Invitrogen, Carlsbad, CA) supplemented with 10% FCS and were added with the indicated doxorubicin concentrations for resistance maintenance.

### MTT assay

Cells were seeded in 96-well plates at a density of 5 x10^3^/well. Cytotoxicity of doxorubicin in the series of resistant MCF-7 sublines, MCF-7/ADR and MCF-7/WT cells was determined by MTT assay after cells were incubated with doxorubicin for 3–5 days.

### RT-PCR

Gene expressions were analyzed by RT-PCR. Total RNA was isolated from a series of MCF-7 cells by TRIzol reagent (Invitrogen, Carlsbad, CA) according to the manufacturer’s instruction. The first strand cDNA was synthesized from extracted RNA using an Oligo dT as the primer. After cDNA was synthesized, the primers of target genes were employed and GoTag Green Master Mix (Promega, Madison, WI) was used to amplify the genes. The gene products were separated by a 2% agarose gel (2% agarose/TAE buffer 100 ml). The DNA was stained by ethidium bromide for 3 minutes, and then detected by UVP BioDoc-IT imaging system (UVP, CA, USA). The primer sequences for RT-PCR were listed in S1 Table.

### Ploidy composition analysis

The nuclear DNA contents of MCF-7/WT, MCF-7/ADR-1024 and MCF-7/ADR cells were determined using flow cytometry. At least 1×10^5^ cells were centrifuged and fixed with ethanol. Cells were resuspended in 500µL of PI-staining buffer (Becton Dickinson, San Jose, CA, USA) for thirty minutes before subjected to flow cytometer. The cells were analyzed immediately using a FACS Caliber (Becton Dickinson, San Jose, CA, USA). The fluorescence was measured with FL2 band-pass filter.

### Western blot analysis

A series of MCF-7 cells were seeded in 6 cm plates at a density of 1x10^6^/well with doxorubicin treatment. After incubation for two days, total proteins were isolated from cells lysates in RIPA buffer which contained 150 mM NaCl, 1.0%(v/v) Triton X-100, 0.5%(v/v) sodium deoxycholate, 0.1% (w/v) SDS, 50 mM Tris (8.0). Protein concentrations were determined by Bradford assay using the BioRad Protein Assay Kit (BioRad; Richmond, CA). Protein samples were separated onto a 10% SDS-polyacrylamide gels for detection of p53, and a 6% gels for P-gp and MRP1. Protein samples were then transferred to an Immobilon NC membrane (Millipore Corp; Bedford, MA) in transfer buffer (25 mM Tris, 190 mM glycine and 20% (v/v) methanol). The membranes were then blocked in 5% milk TBS-T (Tris buffered saline Tween-20) at room temperature for 1 hour. Proteins were labeled with anti-MDR1 (Santa Cruz, CA, USA), anti-MRP1 (Chemicon, Temecula, CA) and anti-p53 (Epitomics Inc. Burlingame, CA). Immunoreactive bands were detected by anti-mouse HRP or anti-rabbit peroxidase-conjugated secondary antibody (Santa Cruz, CA, USA). The protein expression of Nrf-2 in the cytoplasmic and nuclear fractions was determined using BioVision Nuclear/Cytosol Fractionation Kit (Biovision, CA, USA), following the manufacturer’s instruction. Proteins were labeled with anti-Nrf-2 (Santa Cruz, CA, USA). Immunoreactive bands were detected by anti-mouse HRP peroxidase-conjugated secondary antibody (Santa Cruz, CA, USA). Protein were visualized via enhanced chemiluminescence (ECL, detection kit, GE Healthcare, Buckinghamshire, UK) and detected by UVP BioDoc-IT imaging system (UVP, CA, USA).

### Flow cytometry analysis of MRP1 and MDR1 function

Cells were pretreated with or without P-gp inhibitor verapamil 4 μM for 2 hours. After the pretreatment, cells were incubated with 10 μM rhodamine 123 (MDR1 substrate) in dark at 37°C for 1 hour. Then, cells were trypsinized from the subfluent monolayer, and the pellet was washed twice with ice-cold PBS. Intracellular rhodamine 123 accumulation was analyzed immediately using a FACS Caliber (Becton Dickinson, San Jose, CA, USA). The fluorescent of rhodamine 123 was measured by FL1 band-pass filter. MRP1 function was detected as described [6]. Cells were pretreated with or without MRP1 inhibitor MK571 (10 μM) for 2 hours. After the pretreatment, cells were incubated with 0.3 μM calcein-AM in dark at 37°C for 1 hour to yield the MRP1 substrate calcein by the esterase located at the cytoplasm. After calcein accumulated, cells were trypsinized from the subfluent monolayer of cells, and the pellet was washed twice with ice-cold PBS. The cells were analyzed immediately using a FACS Caliber (Becton Dickinson, San Jose, CA, USA). The fluorescent of calcein was measured by FL1 band-pass filter.

### Full-length p53 cDNA sequencing

The primers used for amplifying p53 cDNA fragments, overlapping the full-length p53 coding sequence, were as follows: codons 1–148: 5’-ATGGAGGAGCCGCAGTCA-3’ and 5’-ATCAACCCACAGCTGCACAGGG-3’, codons 118–353: 5’-GGGACAGCCAAGTCTGTGACT-3’ and 5’-CCTGGGCATCCTTGAGTT-3’, codons 253–393: 5’-ACCATCATCACACTGGAAGACTCC-3’ and 5’-ATGTCAGTCTGAGTCAGG-3’. PCR products were sequenced by Misson Biotech (Taipei, Taiwan) as described [8, 31].

### Detection of intracellular ROS levels

Cells were seeded in 6 cm plates at a density of 1x10^6^/well without doxorubicin treatment and were incubated with 5 μM DCFH-DA (Invitrogen, Carlsbad, CA) for 30 min at 37°C before the harvest time point. The cells were then washed and resuspended in FACS buffer (PBS+1% FBS). The intensity of FL1 was measured and the data were analyzed with CellQuest software (BD, San Diego, CA).

### Wound-healing assay

Cells in medium containing 10% FBS were seeded in 24-multiwell plates (Becton Dickinson, San Jose, CA, USA). After cells grew to confluence, wounds were made by sterile pipette tips. Cells were washed with PBS and refreshed with medium with or without 10% FBS. After overnight incubation at 37°C, distances of cell migration were photographed.

### Detection of CD44 high/CD24 low cells by flow cytometry

The series of resistant MCF-7 cells and MCF-7/WT cells were analyzed after staining with CD24-FITC or CD44-PE antibody. At least 1×10^5^ cells were centrifuged at 500 g for 3 min at 4°C and resuspended in 10 µL of PE-conjugated anti-CD44 (Becton Dickinson, San Jose, CA, USA) and 10 µL of FITC-conjugated anti-CD24 (Becton Dickinson, San Jose, CA, USA), then incubated at 4°C in the dark for 30 minutes. Meanwhile, cells were incubated respectively with the isotypes of CD44 and CD24 as negative controls. The labeled cells were washed 3 times and then analyzed by the FACS Caliber (Becton Dickinson, San Jose, CA, USA). Each analysis detected 10,000 cells.

### Colony formation assay

Five thousand cells in 1.5 mL 0.35% agarose-containing growth medium were overlaid with 1.5 mL 0.5% agarose-containing growth medium, and the cells were incubated for 10–14 days. The whole-well images were photographed by digital camera and 3 fields (X 10 magnification) of each well were imaged.

### Statistical analysis

Data are statistically presented as the mean±S.D for the indicated number of separate experiments. Comparisons between groups were analyzed via Student’s *t*-tests. Probability values of P < 0.05 were considered statistically significant.

## Results

### Establishment of a series of MCF-7 cell lines with incremental strength of resistance to doxorubicin

We repeatedly treated the MCF-7/WT with doxorubicin to establish a series of MCF-7 cell lines exerting different strengths of resistance. Resistant cells were selected by doxorubicin starting at 1 nM. After the cells were tolerable, drug concentration was doubled till the cells acquired resistance. Followed by repeated treatments, 11 cell lines were established with incremental strength of resistance to doxorubicin ranging from 1 nM to 1 μM. The series of resistant cells are so-called MCF-7/ADR-n which was treated with n nM of doxorubicin and named as MCF-7/ADR-1, MCF-7/ADR-2, MCF-7/ADR-4, MCF-7/ADR-8, MCF-7/ADR-16, MCF-7/ADR-32, MCF-7/ADR-64, MCF-7/ADR-128, MCF-7/ADR-256, MCF-7/ADR-512 and MCF-7/ADR-1024, respectively. The eleven MCF-7/ADR-n cell lines were cultured in the indicated concentrations to maintain the resistance strength. Cytotoxicity of the 11 MCF-7/ADR-n cell lines was measured by the MTT assay. The IC_50s_ in parent MCF-7 (MCF-7/WT) and authentic doxorubicin-resistant MCT-7 (MCF-7/ADR) are 0.1μM and 12.9 μM, respectively, and the IC_50s_ in the 11 MCF-7/ADR-n cell lines are in between (Table 1). The IC_50_in MCF-7/1024 (10.3 μM) is close to that in MCF-7/ADR. All the MCF-7/ADR-n cell lines have low resistance index except MCF-7/1024 which is under approximate 1 μM doxorubicin selection has the resistance index similar to MCF-7/ADR (93.6 vs 117.2).

**Table 1:** IC_50_ in doxorubicin-resistant MCF-7 cell lines.

**Concentration of doxorubicin (nM)**	**Cell subline**	**IC50 (μM)**	**Resistance index (RI)**
-	MCF-7 /WT	0.110±0.012	1.0
1	MCF-7 /ADR-1	0.117±0.014	1.1
2	MCF-7 /ADR-2	0.140±0.031	1.2
4	MCF-7 /ADR-4	0.145±0.024	1.3
8	MCF-7 /ADR-8	0.170±0.022	1.5
16	MCF-7 /ADR-16	0.215±0.069	1.9
32	MCF-7 /ADR-32	0.275±0.024	2.5
64	MCF-7 /ADR-64	0.366±0.125	3.3
128	MCF-7 /ADR-128	0.388±0.004	3.5
256	MCF-7 /ADR-256	0.575±0.079	5.2
512	MCF-7 /ADR-512	1.720±0.584	15.6
1024	MCF-7 /ADR-1024	10.300±0.509	93.6
1000	MCF-7/ ADR	12.900±1.940	117.2

Parent MCF-7/WT, authentic MCF-7/ADR and 11 MCF-7/ADR-n cell lines were treated with doxorubicin at 1x10^–10^ -1x10^–4^ M. Cell survival was measured using MTT assay, n = 3.

### Gene expression and morphological changes of the MCF-7 /ADR-n cell lines

**Figure 1:**
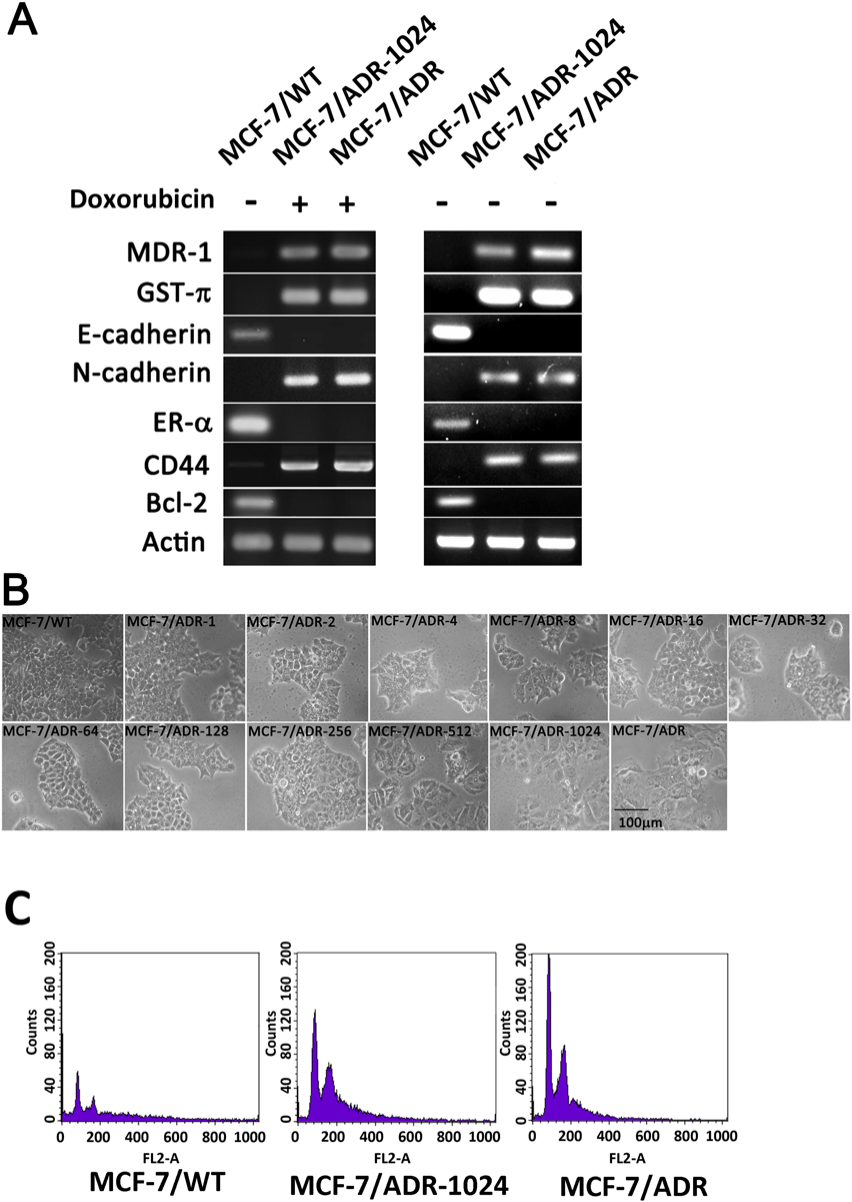
Differential gene expressions and morphological changes of the MCF-7/ADR-n cell lines. (A) Gene expression profile in MCF-7/WT, MCF-7/ADR and MCF-7/ADR-1024. MCF-7/ADR and MCF-7/ADR-1024 were cultured with 1 μM doxorubicin or with doxorubicin deprivation for 14 days. mRNA levels were measured using RT-PCR followed by electrophoresis. (B) Phase-contrast microscopic images of MCF-7/WT, authentic MCF-7/ADR and 11 MCF-7/ADR-n cell lines cultured in media with the indicated doxorubicin concentrations; scale bar = 100 μm. (C) DNA ploidy status. DNA composition was determined by propidium iodide staining in MCF-7/WT, MCF-7/ADR-1024 and MCF-7/ADR cells, using flow cytometry.

The acquisition of drug resistance may be related to gene expression involving in drug transporters expression, anti-apoptosis and detoxification processes. Those characteristics of tumor cells can be regarded as resistant phenotype. To understand if the MCF-7/ADR-1024 exerted resistance phenotype, expression levels were measured for genes involving in ABC transporter proteins, apoptosis, antioxidants and drug metabolism, DNA repair and cell proliferation as described in S2 Table. MCF-7/ADR-1024 and MCF-7/ADR express high levels of drug transporter ABCB1 gene, detoxifying gene GST-π, basal-like gene N-cadherin and cancer stem cell surface marker CD44 gene. On the other hand, MCF-7/ADR-1024 and MCF-7/ADR lose expression of ER-α, E-cadherin and apoptosis-related Bcl-2 genes. Similar expression patterns were shown when MCF-7/ADR-1024 and MCF-7/ADR cultures were deprived from doxorubicin pressure for 2–3 generations (Fig. 1A). The morphological changes of the MCF-7/ADR-n were photographed by contrast image (Fig. 1B). It was noted that the morphology of the series of MCF-7 sublines changed from a cobble stone-like phenotype with strong cell-cell adhesions to a spindle-like appearance, i.e. MCF-7/ADR-1024 which became elongated in shape and were disassociated from their neighboring cells. The ploidy analysis was determined by flow cytometry. It showed that MCF-7/ADR104 and MCF-7/ADR had similar DNA ploidy status in comparison to MCF-7/WT (Fig. 1C). Although the two cell lines authentic MCF-7/ADR and MCF-7/ADR-1024 were established independently, they share genotypic and phenotypic similarities.

### mdr1 gene expressed in MCF-7/ADR-1024 and MRP-1 gene mainly expressed in MCF-7/ADR-128 and MCF-7/ADR-256

**Figure 2:**
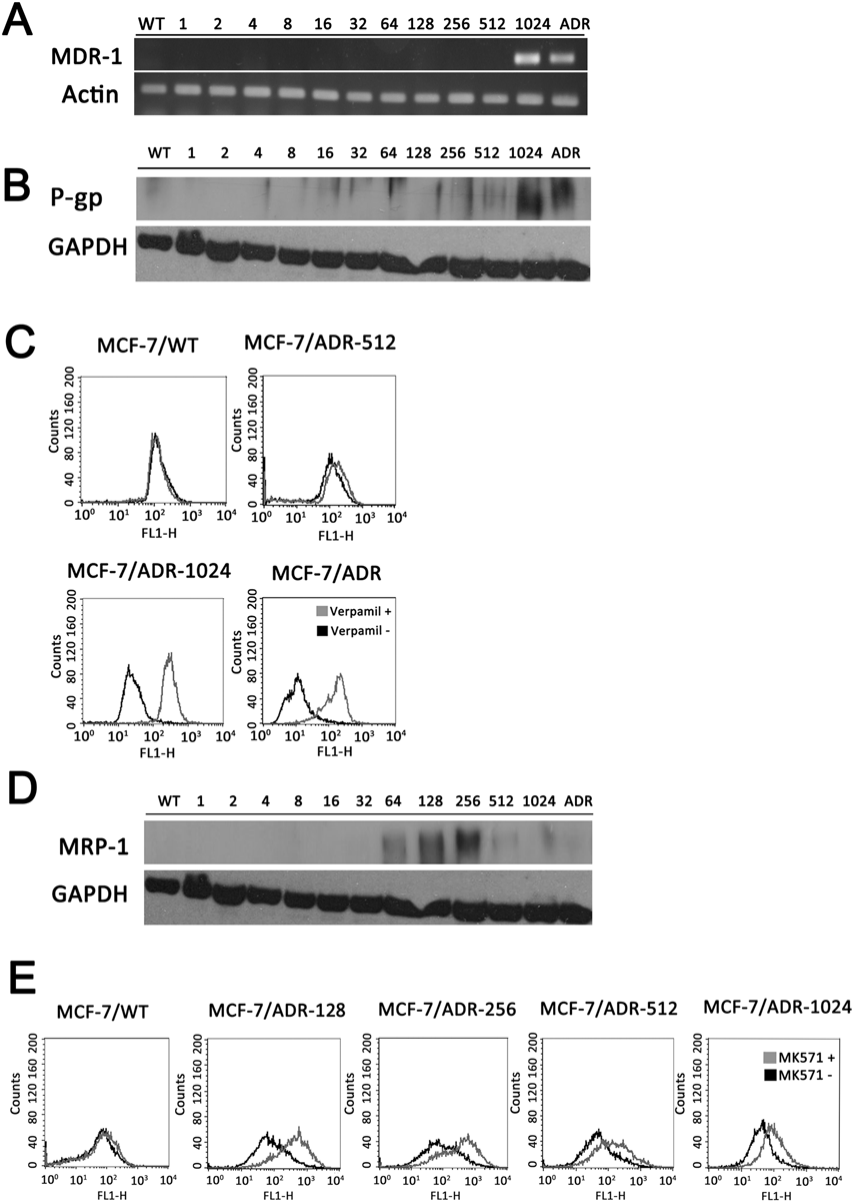
ABC transports are upregulated in the induction of drug resistance. (A)MDR-1 mRNA levels in the series of resistant cells. (B) P-gp detected in MCF-7/ADR-1024 and MCF-7/ADR using Western blotting. (C) P-gp function detected by the accumulation of intracellular rhodamine-123 dye followed by FACS analysis. The cells were treated with or without verapamil 4 μM for 2 hours and then were added with rhodamine-123 dye 5 μM for 1 h. Histogram displays the X-axis as fluorescence intensity and the Y-axis as cell counts. (D) Protein levels of MRP-1 measured by Western blotting. (E) MRP-1 protein function detected by the accumulation of fluorescent dye calcein-AM followed by FACS analysis. Cells were treated with or without MK571 10 μM for 2 hours and then were added with calcein-AM 0.3 μM for 1 h.

Development of multidrug resistance is related to the expression of genes involving in drug transport during chemotherapy. To understand to what extent the drug transporter proteins appear in the induction of resistance, expression of transporter genes mdr1 and MRP-1 were examined in the series of MCF-7/ADR-n cell lines using RT-PCR and Western blots. Results showed that P-gp and MRP-1 appeared under different resistance strengths. The mdr1 gene and P-gp protein were overexpressed only in MCF-7/ADR-1024 (Fig. 2A and 2B) while MRP-1 expression appeared mainly in MCF-7/ADR-128 and MCF-7/ADR-256 and subsided in MCF-7/ADR-512 and MCF-7/ADR-1024 (Fig. 2D). P-gp efflux function was measured by intracellular accumulation of rhodamine 123 using flow cytometry. Low amounts of rhodamine 123 were accumulated in both MCF-7/ADR-1024 and MCF-7/ADR cells which overexpressed P-gp, compared to the parent MCF-7/WT and MCF-7/ADR-512 cells which were P-gp null. The rhodamine 123 accumulation restored in the presence of a P-gp inhibitor verapamil (Fig. 2C). The efflux function of the transporter was in accordance with the appearance of P-gp. Likewise, MRP-1 efflux activity was measured by the accumulation of MRP-1 substrate calcein-AM. There was no difference in intracellular calcein-AM concentration in parent MCF-7/WT and all the MCF-7/ADR-n cell lines. However, calcein-AM increased in the presence of MRP-1 specific modulator MK571 in MCF-7/ADR-128 and MCF-7/ADR-256 cells which expressed high levels of MRP-1 transporter (Fig. 2E). It implies that P-gp may be more important than MRP-1 in end phenotype of doxorubicin resistance.

### Low expression of Bcl-2 in doxorubicin-resistant cells

**Figure 3:**
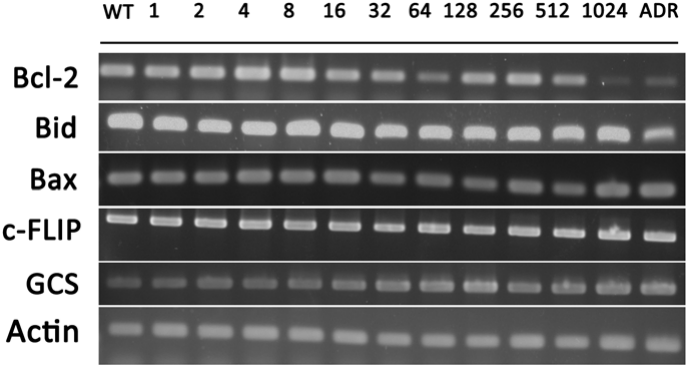
Expression profiles of apoptosis-related genes in the series of resistant cells. mRNA levels of pro-apoptotic and anti-apoptotic genes were detected using RT-PCR in MCF-7/WT, MCF-7/ADR and the series of MCF-7/ADR-n cells.

The chemotherapeutic drug resistance is in some instances associated with an enhanced cellular capacity to avoid apoptosis. Alterations of apoptosis-related proteins have aligned with drug resistance. However, the RT-PCR analysis showed stable gene expression of Bid and Bax in MCF-7/ADR-n cell lines. Bcl-2 was down-regulated in MCF-7/ADR-1024 which highly expressed mdr1 gene (Fig. 3). The mRNA levels of anti-apoptosis regulators such as gucosylceramide synthase (GCS) and FADD-like IL-1β-converting enzyme-inhibitory protein (c-FLIP) did not change among the series of MCF-7/ADR-n cell lines either, although GCS and c-FLIP were thought to participate in drug resistance development. Results indicate that Bcl-2 gene expression may serve as an important apoptosis regulator mediating doxorubicin resistance in MCF-7 cells. It is unclear how the loss of Bcl-2 expression leads to reduce the ability of apoptosis in MCF-7/ADR-1024.

### Repair genes BRCA1/2 and wild type p53 were down-regulated in the development of doxorubicin resistance

**Figure 4:**
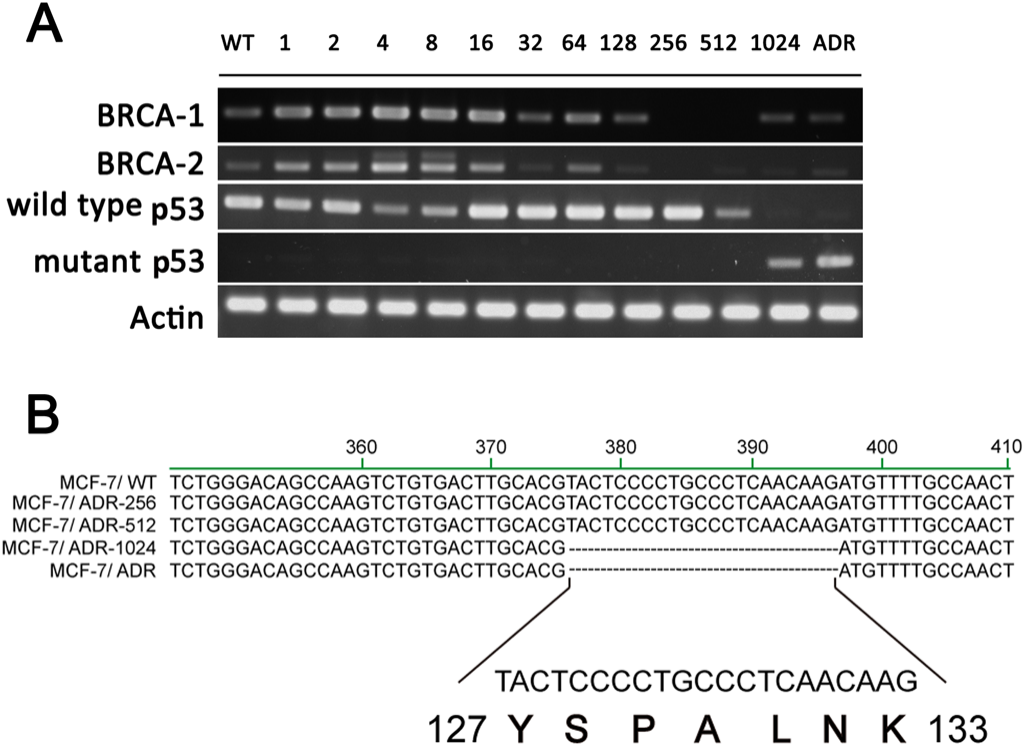
Repair genes BRCA1/2 and wild type p53 are downregulated in the development of doxorubicin resistance. (A)BRCA-1/2 and p53 mRNA levels were detected in the series of MCF-7/ADR-n cells. (B) DNA sequences of the p53 gene. DNAs were extracted from MCF-7/WT, MCF-7/ADR-256 MCF-7/ADR-512 and MCF-7/ADR-1024. Dot lines show a 21 bp deletion at the site of 376–396.

Recent studies suggest that the BRCA1 and BRCA2 gene products may function in the sensing and/or repair of DNA damage involved in drug resistance. Another major repair system is p53 which is related to many drug resistance developments as well. To understand the role of repair genes participating in drug resistance development, we determined expression levels of DNA repair genes in the series of MCF-7/ADR-n cell lines. Results showed that gene expressions of BRCA1 and BRCA-2 were up-regulated in cells with low dose doxorubicin challenges (MCF-7/ADR-1 to MCF-7/ADR-128) whereas the expression began to subside in cells with higher dose doxorubicin treatments (MCF-7/ADR-256 to MCF-7/ADR-1024) (Fig. 4A). In addition, we observed down-regulation of wild type p53 gene and up-regulation of mutant p53 gene in MCF-7/ADR-1024. Several studies suggest that p53 status is associated with breast cancer subtypes. p53 mutations are found frequently in aggressive estrogen receptor (ER)-negative breast cancers, and have been shown to correlate with resistant phenotype. To assess the p53 status in the series of MCF-7/ADR-n cell lines, we sequenced the full-length p53 genes in MCF-7/WT, MCF-7/ADR and MCF-7/ADR-n. Results showed that a deletion was in one allele of the p53 gene at upstream end of exon 5, spanning codons 127–133, in both MCF-7/ADR-1024 and MCF-7/ADR while the 21 bp sequence was conserved in MCF-7/WT, MCF-7/ADR-256 and MCF-7/ADR-512 (Fig. 4B). Accumulation of genetic abnormalities in repair regulator and tumor suppressor seems to be associated with doxorubicin-induced drug resistance.

### Involvement of detoxifying genes Nrf-2, GST-π and PKC-α in doxorubicin-induced resistance

**Figure 5:**
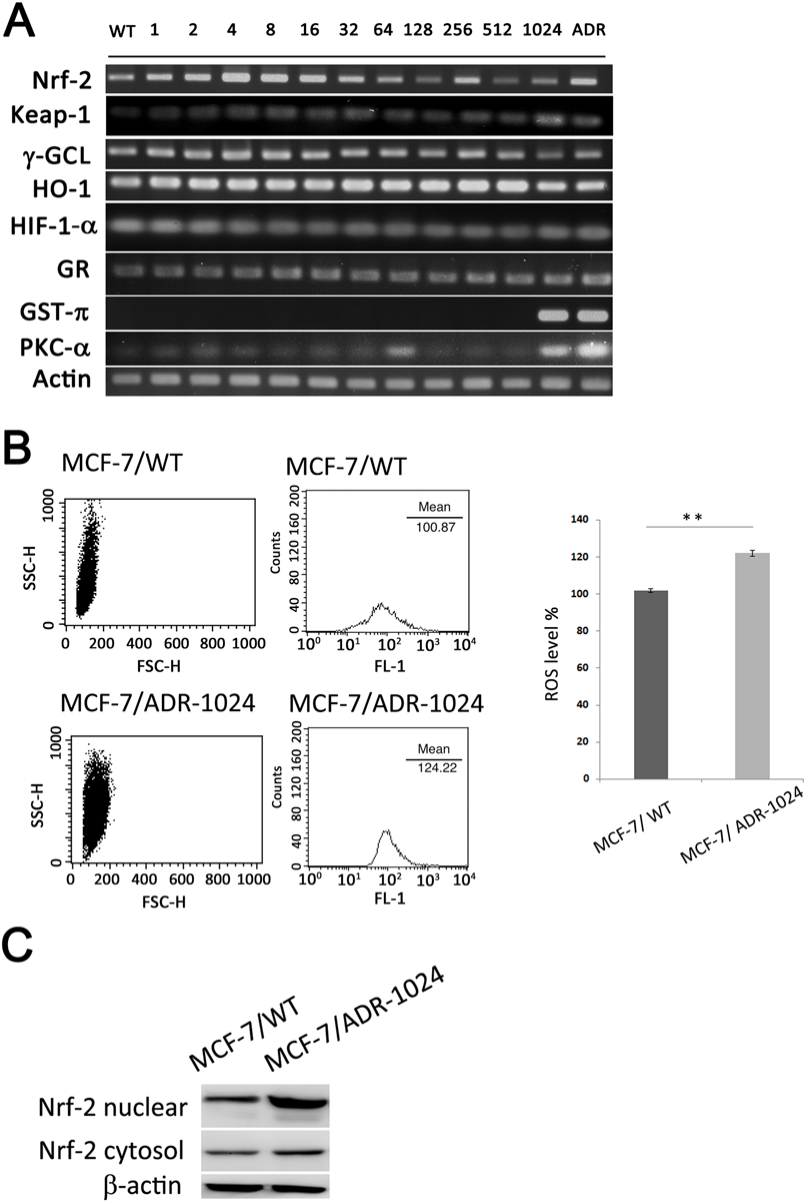
Detoxifying genes Nrf-2, GST-π and PKC-α are associated with doxorubicin-induced resistance. (A) Expression profiles of antioxidation-related genes in the series of MCF-7/ADR-n cells. mRNA levels of Nrf-2, Keap-1, γ-GCL, HO-1, HIF-1-α, GR, GST-π and PKC-α were detected using RT-PCR. (B) Comparison of intracellular ROS generation in MCF-7/WT and MCF-7/ADR-1024. Cells were treated with 50 μM dichlorodihydrofluorescein diacetate (DCF-DA) for 30 minutes. The resulting dichlorofluorescein was measured by flow cytometry. Column bar graph shows mean cell florescence for DCF-DA. Data are expressed as the mean ± S.E.M of three separate experiments (n = 3). **p<0.01. (C) Nrf-2 protein distribution. Nrf-2 proteins in nuclear and cytosolic lysates were determined using Western blotting.

Enhancement of various ROS-scavenging enzymes and redox-sensitive survival machineries in tumor cells had been implicated in chemoresistance and seemed to be associated with poor prognosis. To investigate if the detoxifying genes involving in doxorubicin-induced resistance, we assessed nuclear factor erythroid 2-related factor 2 (Nrf-2) and its downstream genes expression in MCF-7/ADR-n cells. Our data showed no changes in the mRNA levels of Nrf-2, Keap-1, and its downstream cytoprotective genes heme oxygenase-1 (HO-1), gamma-glutamate cysteine ligase (γ-GCL) and glutathione reductase (GR) as well, in the series of MCF-7/ADR-n cell lines. Besides, mRNA levels of hypoxia-inducible factor 1-alpha (HIF-1-α), an oxygen-sensitive transcriptional activator, had no changes, either. In contrast, other important detoxifying enzymes such as glutathione-S transferase-π (GST-π) and protein kinase C-α (PKC-α) were overexpressed in both MCF-7/ADR-1024 and MCF-7/ADR (Fig. 5A). ROS generation was measured by the intracellular intensity of DCF staining. Results showed significant amount of ROS generation in MCF-7/ADR-1024, compared with MCF-7/WT (Fig. 5B). Although no changes in Nrf-2 mRNA levels among the series of MCA-7/ADR-n cell lines, we detected the Nrf-2 protein in cytosol and nucleus and found that large amount of cytosol Nrf-2 protein translocated into nucleus in MCF-7/ADR-1024, compared with MCF-7/WT (Fig. 5C). Overexpression of GST-π, whereby, might be related to the up-stream transcription factor Nrf-2 translocation.

### Epithelial to mesenchymal transition in MCF-7/ADR-1024

**Figure 6:**
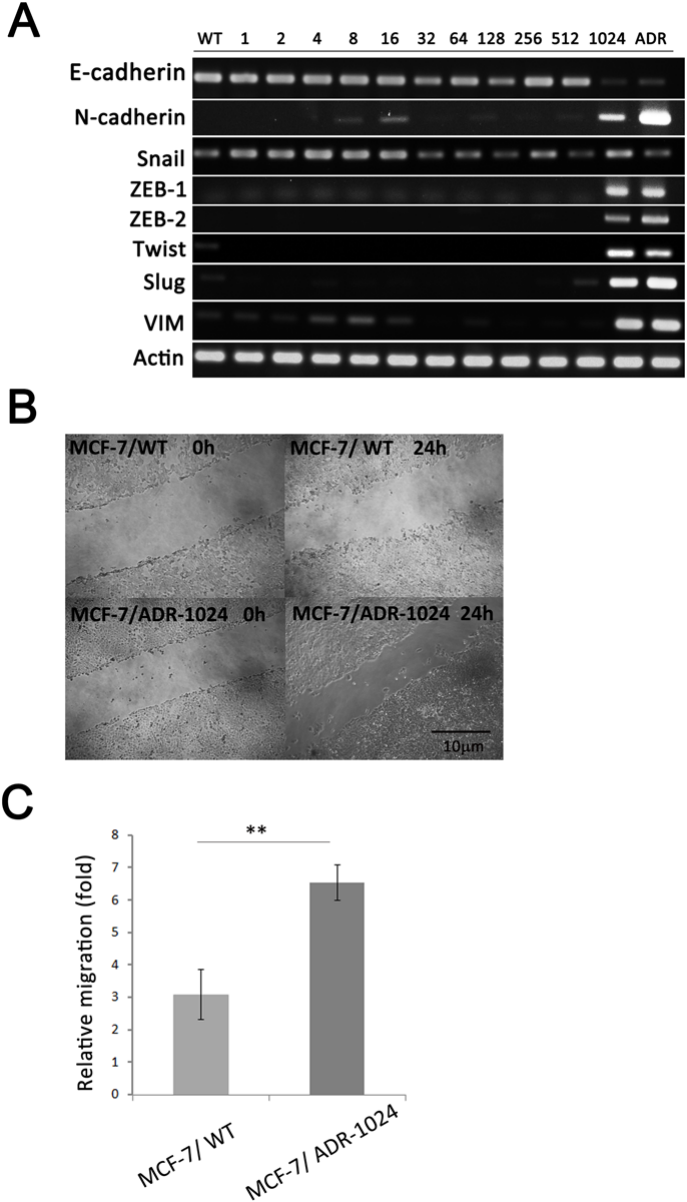
EMT processes in the induction of doxorubicin resistance. (A) EMT-related gene expression profile in the series of MCF-7/ADR-n cell lines. mRNA levels of E-cadherin, N-cadherin, vimentin, ZEB1, ZEB2, Slug, Snail and Twist were detected using RT-PCR. (B) Cell migration of MCF-7/WT and MCF-7/ADR-1024 cells was assessed by wound healing assay. Photographs were obtained at 0 and 24 h to monitor cell migration for the closure of the wound; Scale bar = 10 μM. (C) Graph shows relative cell migration distance of MCF-7/ADR-1024 cells. Data are expressed as the mean±S.E.M of three separate experiments (n = 3). **p<0.01.

Epithelial to mesenchymal transition, characterized by reduced E-cadherin, vimentin and increased N-cadherin expression, had been recognized as a feature of aggressive tumors, associated with regulation of EMT transcriptional factors. There was morphological change from epithelial polarized to mesenchymal/basal when we compared MCF-7/WT with MCF-7/ADR-1024 or MCF-7/ADR. In order to understand the relationship between metastatic phenotype and induction of drug resistance, we studied several transcription factors of EMT in the series of MCF-7/ADR-n sublines. RT-PCR analysis showed that epithelial marker E-cadherin disappeared and mensenchymal markers N-cadherin, ZEB-1/2, Slug, Twist and vimentin were overexpressed in MCF-7/ADR-1024 as well as MCF-7/ADR (Fig. 6A). All other MCF-7/ADR-n sublines did not comply with these gene expression patterns. We also assessed the migration abilities of MCF-7/WT and MCF-7/ADR-1024 and found that MCF-7/ADR-1024 cells with EMT properties had higher migration ability (Fig. 6B, C). It is likely to be a correlation between the invasive phenotypes and the progress toward P-gp transporter-mediated drug resistance.

### Association of PI3K/AKT pathway with EMT properties and drug resistance in MCF-7/ADR-1024

**Figure 7:**
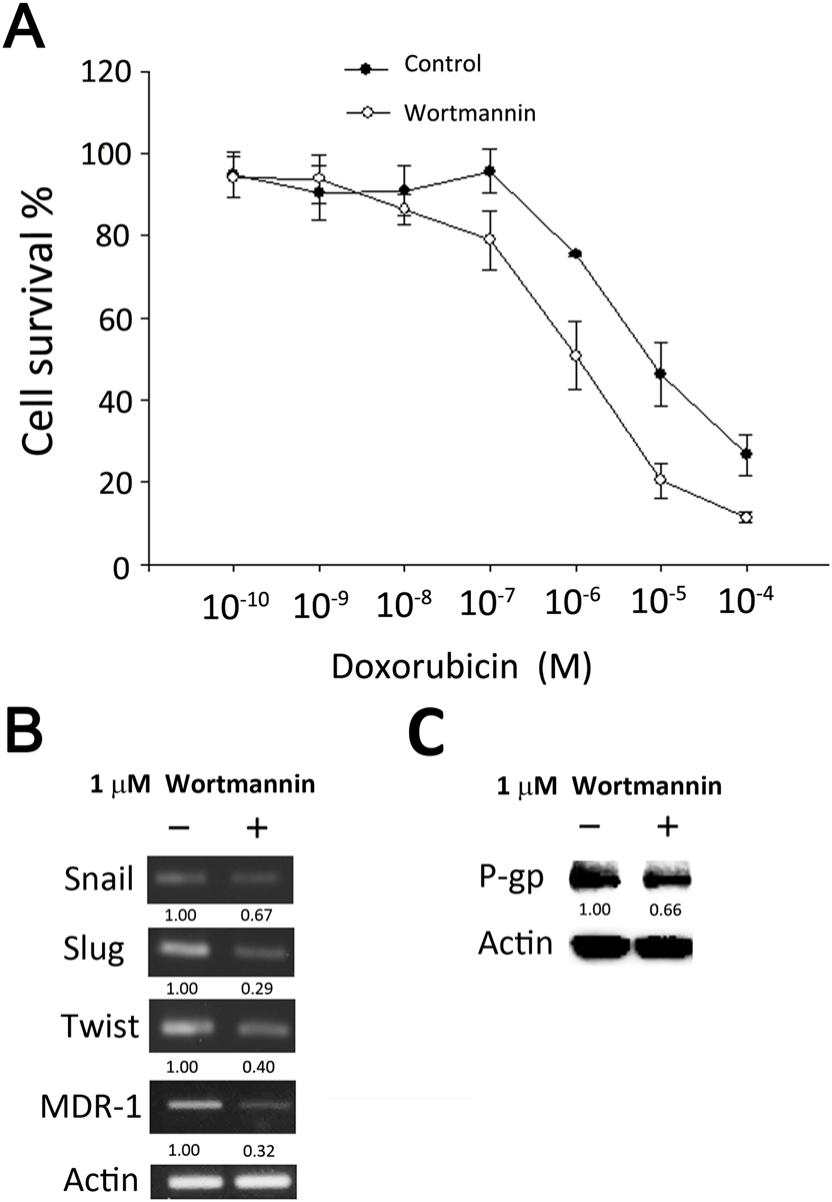
Association of PI3K/AKT pathway with EMT properties and drug resistance. (A) MCF-7/ADR-1024 cell viability was measured in the absence or presence of a PI3K inhibitor wortmannin using MTT assay. (B) PI3K inhibitor decreased the expression of genes related to EMT and drug resistance. MCF-7/ADR-1024 cells were incubated with or without 1 μM of wortmanin for 24 hours. mRNA levels of Slug, Snail, Twist and MDR-1 were determined using RT-PCR. (C) P-gp transporter was suppressed by the PI3K inhibitor. MCF-7/ADR-1024 cells were incubated with or without 1 μM of wortmanin for 24 hours. Protein levels were determined by Western blot.

Recent studies suggest that there may be a cross-talk between EMT and drug resistance. The phosphatidylinositol 3-kinase (PI3K) and AKT pathway is an important mediator in EMT process. MCF-7/ADR-1024 cells carrying drug resistance phenotype were treated with wortmannin, known as a potent PI3K inhibitor, to assess the effect of PI3K/AKT on the development of doxorubicin resistance. In the cell survival assay, IC_50_ of doxorubicin in MCF-7/ADR-1024 cells had a 10 fold decrease when cells were given with additional wortmannin (Fig. 7A). Gene expression levels of EMT transcription factors Slug, Twist and drug resistance marker MDR-1 decreased in the wortmannin treatment (Fig. 7B). Down regulation of PI3K/AKT could suppress P-gp transporter expression as well (Fig. 7C). Blockage of EMT process by targeting the PI3K/AKT signaling may reverse the drug resistance with regard to down-regulation of drug transporter and inhibition of cell proliferation.

### Presence of cancer stem cell-like properties upon doxorubicin selection

**Figure 8:**
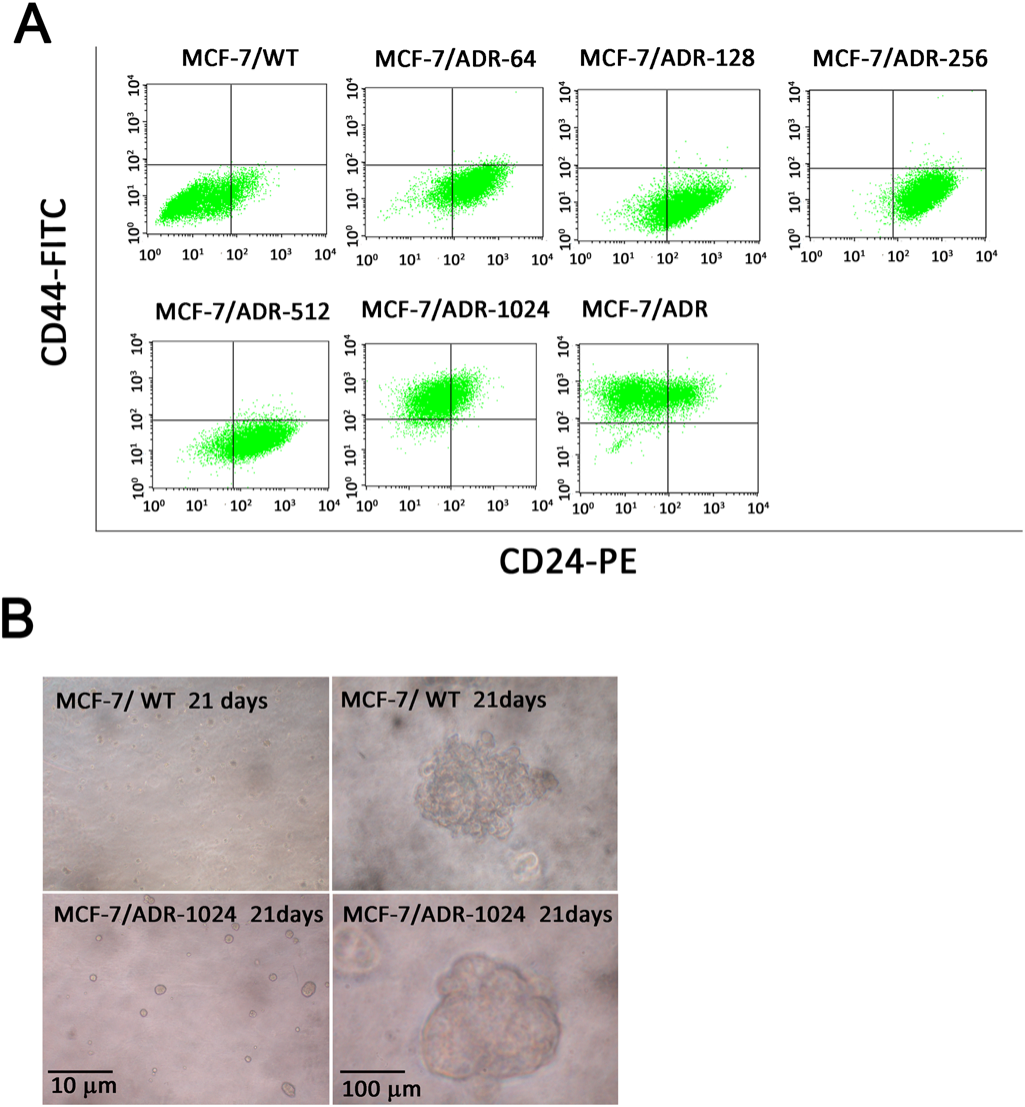
Presence of cancer stem cell-like properties upon doxorubicin selection. (A) Analysis of mammary stem cell markers in selected MCF-7/ADR-n sublines. Cells were sorted for CD44 high/CD24 low cell surface antigens by flow cytometry. Cell population was distributed in the quadrant. (B) Mammospheres formation of MCF-7/ADR-1024 cells in a three-dimensional culture, followed by a limiting dilution assay. A representative phase-contrast microscopic image shows mammosphere formation of MCF-7/ADR-1024 compared to MCF-7/WT from a total of 50 countable  mammospheres on day 21.

Different breast cancer subtypes may result from different breast cancer cell populations. CSCs are defined as a population of cells present in tumors, which express self-renewal, differentiation and resistance. CSCs have been regarded as an important selection factor in cancer progression. To test whether CSC properties were selected in repeated doxorubicin treatments, we examined the stem cell marker CD44 in the series of MCF-7/ADR-n cells. In the flow cytometry analysis, MCF-7/ADR-1024 and MCF-7/ADR were enriched in cells with the CSC-like phenotype (CD44 high/CD24 low) while the other MCF-7/ADR-n sublines did not contain this property (Fig. 8A). We also observed the presence of CD44 low/CD24 high surface marker in cells including MCF-7/ADR-64, MCF-7/ADR-128, MCF-7/ADR-256 and MCF-7/ADR-512, which were under the intermediate doxorubicin challenges. MCF-7/ADR-1024 cells, with CD44 high/CD24 low surface marker expression, formed mammospheres and proliferated in three-dimensional cultures (Fig. 8B). As mechanisms of drug resistance development were complicated, the heterogeneity of cancer cells or gain of CSC-like properties at least happened to the MCF-7 cells treated with exponential concentrations of doxorubicin.

## Discussion

Induction of drug resistance during chemotherapy is one of the major obstacles to successful treatment of cancers in clinic. Several hypotheses are thought to explain the mechanisms of drug resistance development. Induction and activation of efflux transporter proteins, facilitated detoxification of drugs, alterations in target molecules, enhanced repair of DNA and changes in genes for proliferation or apoptosis have been associated with drug resistance [21–23]. To understand the effect of doxorubicin on the development of drug resistance in tumors, we gradually treated the MCF-7 cells with exponential concentrations of doxorubicin and established eleven resistant MCF-7 sublines which exerted incremental strength of resistance to doxorubicin. Using these eleven resistant MCF-7 sublines, gene expression profiles can be examined not only in the initiation (wild type MCF-7) and the last stage (authentic MCF-7/ADR) of drug resistance but also in the midway of drug resistance progression. In this report, the expression profiles of MDR-related genes have been summarized with regard to the relative intensity among the series of MCF-7/ADR-n cell lines (Table 2).

**Table 2:** Expression of mutidrug resistance-related genes in the series of MCF-7/ADR-n cell lines.

	**MCF-7 / WT**	**ADR-1**	**ADR-2**	**ADR-4**	**ADR-8**	**ADR-16**	**ADR-32**	**ADR-64**	**ADR-128**	**ADR-256**	**ADR-512**	**ADR-1024**	**MCF-7/ ADR**
**MDR-1**	1.0	0.8	0.9	0.7	0.7	0.9	1.3	1.8	1.7	1.1	1.4	8.3	6.6
**MRP-1**	1.0	0.8	0.9	1.1	1.9	1.2	1.9	3.7	6.8	6.3	1.8	1.4	1.7
**Bcl-2**	1.0	0.8	0.8	1.0	0.9	0.8	0.7	0.5	0.8	0.8	0.7	0.1	0.2
**Bid**	1.0	0.7	0.7	0.7	0.7	0.8	0.7	0.7	0.9	0.7	0.8	0.7	0.6
**Bax**	1.0	0.7	0.6	0.7	0.7	0.8	0.6	0.7	0.7	0.7	0.6	0.8	1.1
**c-FLIP**	1.0	0.7	0.7	0.7	0.7	0.8	0.7	0.8	0.8	0.8	0.9	1.1	1.1
**GCS**	1.0	1.2	1.3	1.2	1.5	1.9	2.2	2.1	2.5	1.9	2.3	1.9	1.9
**BRCA-1**	1.0	2.0	2.0	2.8	2.5	2.2	1.1	1.5	0.8	0.2	0.2	0.3	0.3
**BRCA-2**	1.0	1.8	1.8	2.9	2.6	1.8	0.6	1.1	0.4	0.2	0.3	0.2	0.2
**wild type p53**	1.0	0.9	0.8	0.5	0.7	1.1	1.2	1.1	1.1	1.1	0.5	0.1	0.1
**mutant p53**	1.0	1.2	1.3	1.7	1.9	2.0	2.2	1.9	2.3	2.4	2.5	4.9	7.1
**Nrf-2**	1.0	0.8	0.9	1.2	1.1	1.0	0.9	0.7	0.5	0.8	0.5	0.5	1.1
**Keap-1**	1.0	1.1	1.1	1.0	1.3	1.3	1.6	1.3	1.2	1.1	1.2	1.5	1.5
**γγ-GCL**	1.0	0.9	0.9	0.9	0.9	0.8	0.9	0.8	0.7	0.8	0.8	0.5	0.7
**HO-1**	1.0	0.9	0.9	0.8	0.8	0.8	1.1	1.0	1.0	0.9	1.2	0.7	0.8
**HIF-1-αα**	1.0	0.7	0.7	0.5	0.5	0.5	0.7	0.7	0.6	0.5	0.5	0.6	0.9
**GR**	1.0	0.9	0.9	0.9	1.2	1.3	1.4	1.3	1.4	1.3	1.7	1.4	2.2
**GST-ππ**	1.0	0.6	0.5	0.4	0.4	0.4	0.6	0.4	0.4	0.3	0.4	6.7	7.7
**PKC-αα**	1.0	1.1	1.6	1.1	1.1	1.5	1.9	3.1	1.7	1.6	2.0	4.2	6.1
**E-cadherin**	1.0	1.0	0.8	0.9	1.0	0.8	0.6	0.8	0.6	0.9	0.8	0.2	0.2
**N-cadherin**	1.0	0.9	0.8	0.9	1.5	1.6	0.8	0.8	0.7	1.0	0.8	4.8	16.6
**Snail**	1.0	1.8	1.5	2.2	1.9	1.7	0.8	0.9	0.6	1.2	0.6	1.4	0.8
**ZEB-1**	1.0	0.7	1.0	0.9	1.2	1.4	1.1	2.1	0.6	0.5	0.5	2.6	4.0
**ZEB-2**	1.0	1.4	1.3	1.1	1.3	1.1	1.2	1.0	1.0	1.1	0.9	3.4	6.0
**Twist**	1.0	1.2	1.0	1.2	1.2	1.1	1.1	1.0	1.1	1.1	1.1	7.2	6.3
**Slug**	1.0	0.5	0.5	0.8	0.6	0.6	0.5	0.7	0.6	0.7	1.3	8.0	11.5
**VIM**	1.0	1.0	0.6	1.3	1.6	1.0	0.5	0.8	0.5	0.6	0.6	4.3	5.2

Relative mRNA levels are showned as indicated when the expression in MCF-7/WT is regarded as 1.0. The MCF-7/ADR-n cell lines are presented as the abbreviated ADR-n in this table.

**Figure 9:**
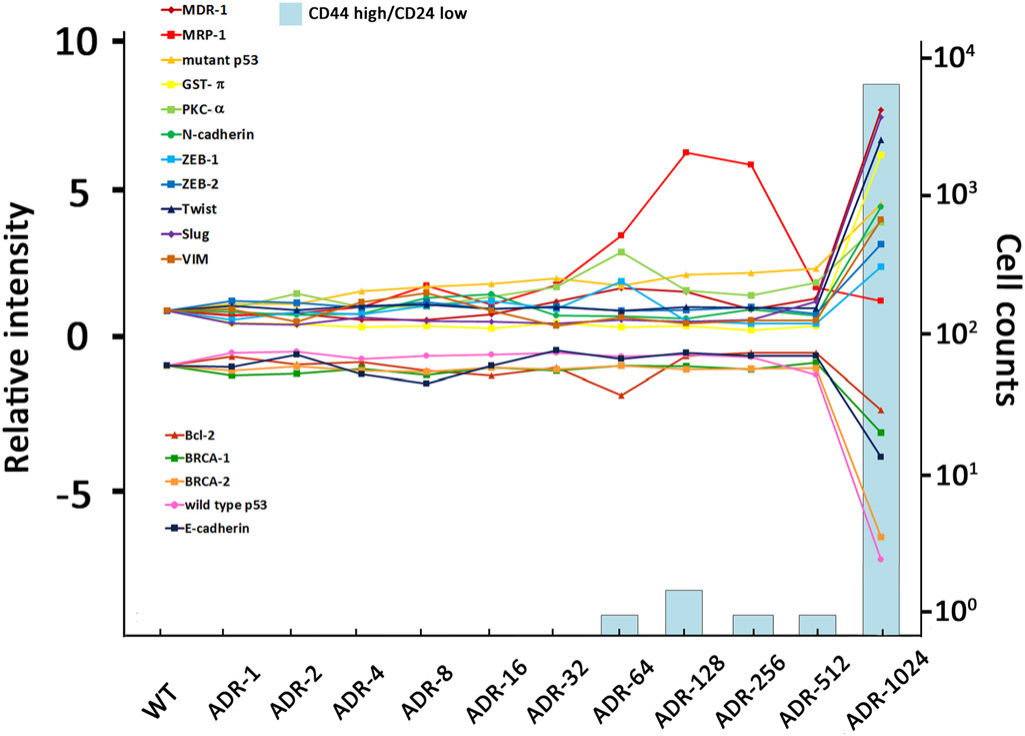
Up/down regulation of multidrug resistance-related genes in the time line of MCF-7/ADR-1024 development. Curves show mRNA levels relative to MCF-7/WT mRNA. X-axis on the left is relative intensity. Positive scales represent increased expression. Negative scales represent decreased expression with a reciprocal conversion. X-axis on the right is the cell counts of CD44 high/CD24 low population.

Amplification of the ABC transporter ABCB1 has been described as a hallmark of MDR in the MCF-7/ADR cells [3, 4]. In this study, we assessed expression levels of ABC transporter ABCB1 in the series of MCF-7/ADR-n cell lines and found that both mRNA and protein of ABCB1 were not observed until the doxorubicin selection dose up to 1024 nM. The MCF-7/ADR-1024 presents similar expression patterns to the authentic MCF-7/ADR which is known to express P-gp [32], detoxifying GST-π [33], gain the basal cell-like phenotype and lose apoptosis abilities [32, 34, 35]. Besides, the process of drug resistance induction accompanies activation of anti-oxidation pathway [36], loss of tumor suppressors function [37] and expression of stem cell marker CD44 [38, 39]. Interestingly, among the series of MCF-7/ADR-n cell lines, differential expressions in most genes were founded only in the last stage with 1024 nM doxorubicin challenge (Fig. 9). During establishment of the series of MCF-7/ADR-n cell lines, cell number in MCF-7/ADR-512 culture dramatically reduced under 1024nM doxorubicin challenge. Only a small number of cells survived in this condition. Meanwhile, the period of transformation from MCF-7/ADR-512 culture into MCF-7/ADR-1024 has increased by more than three times, compared to the other MCF-7/ADR-n sublines. It implies that the MCF-7/ADR-1024 may have originated from a small population which contains MDR characteristics such as expressions of ABC transporter and GST-π. However, we observed that the minor attenuation in repair proteins BRCA-1/BRCA-2 and wild type p53 started at an earlier stage of cells such as MCF-7/ADR-256 or MCF-7/ADR-512. Changes in the expressions of repair proteins BRCA-1/BRCA-2 and wild type p53 are regarded as the contributors for MDR development [40]. The mechanisms of drug resistance development may be operated within proliferating MCF-7 populations to generate phenotypic diversity continuously [41]. Thus, genetic heterogeneity could be suggested among the series of MCF-7/ADR-n cell lines.

Moreover, the tumor initiating and chemotherapy-resistant abilities of CSCs are attributed to the expression of ABC transporters. Long-term exposure of MCF-7 cells to doxorubicin not only selects for cells with a drug-resistant phenotype but also induces a CSC phenotype in a population of the cells [7, 25]. In flow cytometery analysis, we found that cell population was shifted from less stem cell marker region CD44 low/CD24 high to more stem cell marker region CD44 high/CD24 low. The population distribution shift was only observed in the stage from MCF-7/ADR-512 to MCF-7/ADR-1024 culture. The MCF-7/ADR-1024 cells not only express the drug resistant phenotype but also induce a stem cell-like surface marker gene expression and form mammospheres. Recently, EMT markers and mesenchymal phenotype have also been correlated with CSC-like properties [38]. Our data revealed that the series of resistant MCF-7 cell lines exerted more mesenchymal properties under high dose doxorubicin selection and were accompanied with increased expression of stem cell marker CD44. Several signal transduction pathways involved in the regulation of stem cell self-renewal have also been associated with EMT and chemoresistance. Our data are consistent to the study from Saxena *et al.* that the presence of EMT properties within a cell is a prerequisite for up-regulating ABC transporters in response to drugs [4].

We also observed largely increased expression of transporter gene MRP-1 in MCF-7/ADR-128 and MCF-7/ADR-256. Treatment of human small cell lung cancer (SCLC) cell with doxorubicin at 50 nM increased c-jun N-terminal kinase (JNK) activity and simultaneously induced a markedly increase in MRP1 expression [30]. The JNKs, also called the stress-activated protein kinase (SAPK), are serine/ threonine kinases that belong to the mitogen-activated protein kinase (MAPK) family. The JNKs are partly regulated by the protein–protein interactions with GST-π and activated in response to many stressful stimuli including heat shock, UV irradiation and inflammatory cytokines [42, 43]. The presence of MRP-1 and the GST-π could be due to activation of upstream regulators of JNK, inactivation or down-regulation of the phosphatase acting on JNK [6, 33]. In this study, MRP-1 expression subsides in MCF-7/ADR-512 and MCF-7/ADR-1024. The role of the fluctuated amount of MPR-1 is still obscure during the progression of doxorubicin-induced MDR in MCF-7 cells. Regarding the results, the MRP-1 seems to be not a key driving force toward MDR induced by doxorubicin.

DNA repair pathways not only protect the genome against mutations but also act as an adaptive mechanism to promote drug resistance. The p53 is involved in regulation of mdr1 gene. Wild type p53 can inhibit mdr1 gene expression while mutant p53 enhances mdr1 gene expression [44, 45]. The anti-proliferative and repair actions of p53, however, can be altered by loss of p53 alleles, cytoplasmic sequestration or point mutations [46]. Mutant p53 can act as oncoproteins that immortalize and transform primary cells [47]. DNA sequencing data show that MCF-7/ADR-1024 exclusively contains a mutant p53 gene with a deletion at codons 127–133 whereas the other MCF-7/ADR-n cell lines and the parent MCF-7/WT contain wild-type p53 gene. It is interesting to be noted that the identical deletion is resided in the authentic MCF-7/ADR which was established independently by doxorubicin selection [48]. The stability of the 21 bp deletion-containing mutant p53 reduced when the authentic MCF-7/ADR was treated with MDR-reversing agent [31]. The 21 bp deletion in p53 may arise from a point mutation, resulting in abnormal splicing transcript. Point mutations in the conformational domain of p53 can cause abnormal transcripts. For example, G-A transition mutation in p53 was found in MCF-7/ADR cells [18]. This G-A point mutation which comprises a splice acceptor site may alter the splicing pattern, leading to a 21 bp-deleted p53. This G-A transition mutation was reported in other malignant cells as well [19]. However, this hypothesis needs further investigation. In addition, a decrease in Bcl-2 gene expression was found in both MCF-7/ADR-1024 and MCF-7/ADR cells. This phenomenon is consistent to the results of previous studies [18, 49]. Down-regulation of Bcl-2 is involved in the nuclear localization of mutant p53 in MCF-7/ADR cells [50, 51]. Thus, the mutant p53 apparently plays a role to mediate MDR in MCF-7/ADR cells. It would be interesting to study the effect of the unique 21 bp-deleted p53 on gain-of-resistance by construction of a clone stably expressing the 21 bp-deleted p53 in the future.

Doxorubicin increases reactive oxygen species (ROS) production and stimulates protein degradation such as caspase-3 and the ubiquitin-proteasome pathway. There were many lines of evidence that Nrf-2 level was positively correlated with doxorubicin resistance in cancer cell lines [43, 52]. However, in this study, there were no significant changes in Nrf-2 transcription levels among the series of MCF-7/ADR-n cell lines but a notable amount of Nrf-2 was in the nucleus of MCF-7/ADR-1024. Translocation of Nrf-2 from cytosol to nucleus may be associated with doxorubicin stress in MCF-7/ADR-1024 cells. Adaptive activation of the Nrf-2 system may in part participate in the development of acquired resistance to doxorubicin and lead to drive the antioxidant machineries such as GST-π and PKC-α.

Drugs affecting signal transduction pathways can be due to the regulation of the upstream of EMT such as TGF-β signaling and PI3K/AKT pathway [53, 54]. Recent studies suggest that mTOR plays an important role in PI3K/AKT-mediated signaling for cancer stem cell self-renewal and resistance to chemotherapy or radiotherapy [54]. It is thought to be the root cause of treatment failure, cancer recurrence and activation of metastatic activity. Several compounds such as curcumin, resveratrol, silibinin and ondole 3-carbinol have been reported to regulate PI3K/AKT pathway in many cells [54, 55]. Our results showed that inhibition of PI3K/AKT by wortmanin partially suppressed the expression of EMT transcription factors Slug, Twist and drug resistance marker MDR-1 in MCF-7/ADR-1024. P-gp transporter was down-regulated as well. It implicates that the increase of doxorubicin resistance in MCF-7/ADR-1024 may be in part associated with PI3K/AKT signaling and the EMT properties, resulting in the increase of P-gp transporter function. Further study should be investigated to support the hypothesis.

**Figure 10:**
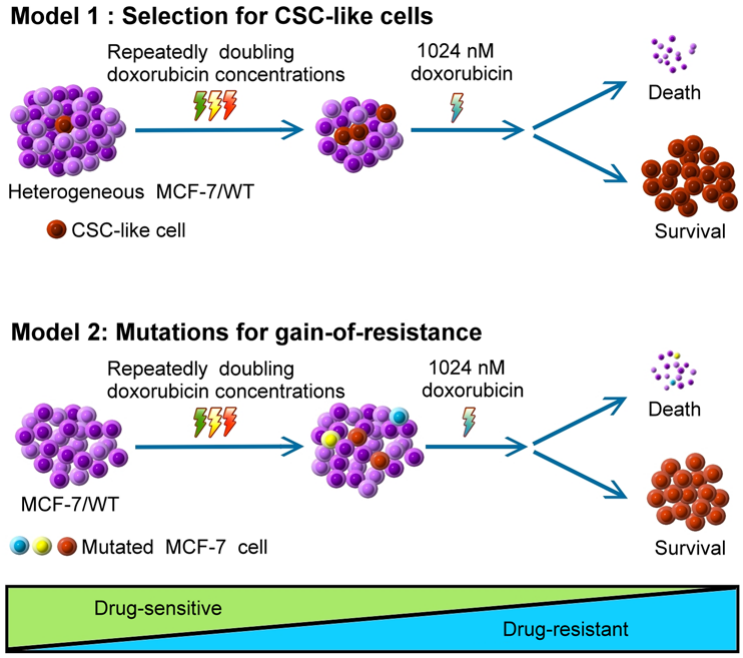
Two models illustrate development of doxorubicin resistance. Model 1: Selection for CSC-like cells. Wild type MCF-7 cells are heterogeneous. Only when cells suffer from high stringent 1024 nM doxorubicin challenge, the cells with CSC-like property survive. Model 2: Mutations for gain-of-resistance. Mutations occur upon repeated treatments with a double concentration doxorubicin. A small population of mutated cells are allowed to gain resistance and survive from 1024 nM doxorubicin challenge.

Taken together, mechanisms of doxorubicin resistance development could be described by two possible models according to our results (Fig. 10). Model 1: Wild type MCF-7 cancer cells may be heterogeneous. The majority of MCF-7/WT cells are non-invasive epithelial cells and are sensitive to exponential concentrations of doxorubicin. A small population of cells may be alive due to doxorubicin-induced activation of antioxidants, drug metabolism, DNA repair and cell proliferation pathways in these cells. That allows cells to be transformed to mesenchymal type with basal cell-like properties. The EMT was thought to enhance the generation of cancer stem cell-like cells [4, 26]. These CSC-like cells have the ability to up-regulate the expression of ABC transporters, resulting in MDR. Cell number of this MDR-presenting population may be so small as to be undetectable in low dose doxorubicin-treated cultures. When doxorubicin selection dose is higher than 1 μM, it may be a catastrophe to the MCF-7/ADR-512 cell line which does not express P-gp in most cells. Only the MDR-presenting population survives and propagates under such high stringent condition. Model 2: Mutations allow cancer cells to gain resistance and survive from stresses or toxins. After a long term treatment with chemotherapeutic drugs, cancer cells accumulated lots of genotoxic damages to cause the loss of expression of the tumor suppressors [8, 56]. In our study, a 21 bp deletion in p53 gene may render the tumor suppressor loss of function and gain of drug resistance in MCF-7/ADR-1024. Besides, accumulation of genotoxic damages also possibly cause mutations in genes involving in signal transduction pathways. Regulation of cell survival, proliferation and differentiation is thereby associated with chemoresistance. Model 2 can explain why cancers that tend to initially respond to chemotherapy show much aggressive and MDR phenotype on relapse. Either model 1 or model 2 requires doxorubicin dose approaching 1 μM to alter gene regulation. A large scale gene profiling via either conventional RT-PCR or microarrays could be employed to reveal more information of the genes conveying programmable regulations in the development of MDR.

## Supporting Information

S1 Table Primer sequences for RT-PCR. Primers were designed and verified using Primer3web (http://primer3.ut.ee/) and on-line Primer blast (http://www.ncbi.nlm.nih.gov/tools/primer-blast/).
(DOCX)

S2 Table Drug-resistance-related genes screened in this study. (DOCX)
