# Supplementary material for: A Critical Dose of Doxorubicin Is Required to Alter the Gene Expression Profiles in MCF-7 Cells Acquiring Multidrug Resistance
Source: PLoS One. 2015 Jan 30;10(1):e0116747. doi: 10.1371/journal.pone.0116747 (PMC4312059; doi:10.1371/journal.pone.0116747)
Supplement: S1 Table — Primers were designed and verified using Primer3web (http://primer3.ut.ee/) and on-line Primer blast (http://www.ncbi.nlm.nih.gov/tools/primer-blast/). (DOCX) [file pone.0116747.s001.docx]

# Supporting Information Legends

**Table S1. Primer sequences for RT-PCR**

|  | **Sequence** | | **Size** |
| --- | --- | --- | --- |
| **MDR-1** | Forward | 5'- CCCATCATTGCAATAGCAGG -3' | 157 |
|  | Reverse | 5'- GTTCAAACTTCTGCTCCTGA -3' |  |
| **GST-π** | Forward | 5'- CTCCGCTGCAAATACATCTC -3' | 137 |
|  | Reverse | 5'- ACAATGAAGGTCTTGCCTCC -3' |  |
| **CDH-1** | Forward | 5'- TCACAGCAGAACTAACACACGGG -3' | 165 |
|  | Reverse | 5'-GTGGTCACTTGGTCTTTATTCTGGTTATCC-3' |  |
| **CDH-2** | Forward | 5'- AATGACAATCCTCCAGAGTTTACTGCC -3' | 210 |
|  | Reverse | 5'- GGTGACTAACCCGTCGTTGCT -3' |  |
| **ER-α** | Forward | 5'- CAGGCACATGAGCAACAAAG -3' | 81 |
|  | Reverse | 5'- TCCAGCAGCAGGTCGTAGAG -3' |  |
| **CD44** | Forward | 5'-TTTGCATTGCAGTCAACAGTC-3' | 234 |
|  | Reverse | 5'-GTTACACCCCAATCTTCATGTCCAC-3' |  |
| **Bcl-2** | Forward | 5'-CAGAATAACCAGAACTAAGGGTATGAAGGA-3' | 159 |
|  | Reverse | 5'-CCAAACGGAGCTGCACT-3' |  |
| **β-actin** | Forward | 5'- ACAGCTGAGGGAAATCGTGGG -3' | 150 |
|  | Reverse | 5'- ACTTGCGCTCAGGAGGAGCAATG -3' |  |
| **Bid** | Forward | 5'- GCATGTCAACAGCGTTCCTA -3' | 222 |
|  | Reverse | 5'- GGAACCTGCACAGTGGAAAT -3' |  |
| **Bax** | Forward | 5'- GGGGACGAACTGGACAGTAA -3' | 122 |
|  | Reverse | 5'- CAGTTGAAGTTGCCGTCAGA -3' |  |
| **c-FLIP** | Forward | 5'- TTCCAGGCTTTCGGTTTCTT -3' | 443 |
|  | Reverse | 5'- GTCCGAAACAAGGTGAGGGT -3' |  |
| **GCS** | Forward | 5'- CCTTTCCTCTCCCCACCTTCCTCT -3' | 302 |
|  | Reverse | 5'- GGTTTCAGAAGAGAGACACCTGGG -3' |  |
| **BRCA-1** | Forward | 5’-CAGGCACATGAGCAACAAAG-3’ | 240 |
|  | Reverse | 5’-TCCAGCAGCAGGTCGTAGAG-3’ |  |
| **BRCA-2** | Forward | 5’-AGAGTCCCTGGTGTGAAGCAAG-3’ | 329 |
|  | Reverse | 5’-GACAGCGCAGAAGTGAGCATC-3’ |  |
| **Wild type p53** | Forward | 5'- GAAGACCCAGGTCCAGATGA -3' | 228 |
|  | Reverse | 5'- CTTGTTGAGGGCAGGGGAGTA -3' |  |
| **Mutant p53** | Forward | 5'- GAAGACCCAGGTCCAGATGA -3' | 222 |
|  | Reverse | 5'- TGGCAAAACATCGTGCAAGTC -3' |  |
| **Nrf-2** | Forward | 5'- TCACCATCTCAGGGGGAG -3' | 770 |
|  | Reverse | 5'- CAACATACTGACACTCCAATGC -3' |  |
| **Keap-1** | Forward | 5'-CAGCCAAGGTCCCTGAGT-3' | 219 |
|  | Reverse | 5'-GTGTAGCTGAAGGTGCGGT-3' |  |
| **γ-GCL** | Forward | 5'- AGAGAAGGGGGAAAGGACAA -3' | 231 |
|  | Reverse | 5'- GTGAACCCAGGACAGCCTAA -3' |  |
| **HO-1** | Forward | 5'-CAGAAGAGCTGCACCGCAAG-3' | 309 |
|  | Reverse | 5'-GGTAGAGCTGCTTGAACTTG-3' |  |
| **HIF-1-α** | Forward | 5'-ACTTCTGGATGCTGGTGA-3' | 1154 |
|  | Reverse | 5'-CGGTGGGTAATGGAGACA-3' |  |
| **GR** | Forward | 5'-TCTAAGACATCACTGATG-3' | 324 |
|  | Reverse | 5'-GAATTCGTCTACGATGAT-3' |  |
| **PKC-α** | Forward | 5'- ACTCCACGCGTCTCAGGA -3' | 101 |
|  | Reverse | 5'- GCGCGCGATGAATTTGTG -3' |  |
| **Snail** | Forward | 5'- GAA AGGCCTTCAACTGCAAA -3' | 249 |
|  | Reverse | 5'- TGACATCTGAGTGGGTCTGG -3' |  |
| **ZEB-1** | Forward | 5'- GATGATGAATGCGAGTCAGATGC -3' | 86 |
|  | Reverse | 5'- ACAGCAGTGTCTTGTTGTTGTAG -3' |  |
| **ZEB-2** | Forward | 5'- AACAACGAGATTCTACAAGCCTC-3' | 176 |
|  | Reverse | 5'- TCGCGTTCCTCCAGTTTTCTT-3' |  |
| **Twist** | Forward | 5'- GGAGTCCGCAGTCTTACGAG -3' | 201 |
|  | Reverse | 5'- TCTGGAGGACCTGGTAGAGG -3' |  |
| **Slug** | Forward | 5'- AGATGCATATTCGGACCCAC -3' | 257 |
|  | Reverse | 5'- CCTCATGTTTGTGCAGGAGA -3' |  |
| **VIM** | Forward | 5'- GGAAGCTGCTGGAAGGCGA -3' | 159 |
|  | Reverse | 5'- CCTGTCCATCTCTAGTTTCAACCGTCTTA -3' |  |
