# Supplementary material for: A Critical Dose of Doxorubicin Is Required to Alter the Gene Expression Profiles in MCF-7 Cells Acquiring Multidrug Resistance
Source: PLoS One. 2015 Jan 30;10(1):e0116747. doi: 10.1371/journal.pone.0116747 (PMC4312059; doi:10.1371/journal.pone.0116747)
Supplement: S2 Table — (DOCX) [file pone.0116747.s002.docx]

**Table S2. Drug-resistance-related genes screened in this study**

| **Category** | **Gene** | **Description** |
| --- | --- | --- |
| **Transporter** | **MDR-1** | Multidrug resistance protein 1 (MDR-1) is an ATP-dependent drug efflux pump for xenobiotic compounds with broad substrate specificity. It is responsible for decreased drug accumulation in multidrug-resistant cells and often mediates the development of resistance to anticancer drugs. |
|  | **MRP-1** | Multidrug resistance-associated protein-1 (MRP-1) is an ATP-dependent drug efflux pump, and functions as a multispecific organic anion transporter. |
| **Apoptosis** | **Bcl-2** | Bcl-2 is the founding member of the Bcl-2 family, regulates cell death by mediating anti-apoptotic and pro-apoptotic proteins. |
|  | **Bid** | Bid is a mediator of mitochondrial damage induced by caspase-8 |
|  | **Bax** | Bax forms a heterodimer with Bcl-2, and functions as an apoptotic activator. |
|  | **c-FLIP** | The protein encoded by c-FLIP is a regulator of apoptosis and is structurally similar to caspase-8. |
|  | **GCS** | Glucose ceramide (GlcCer) is generated with glycosylation of ceramide by glucosylceramide synthase (GCS) , then cell apoptosis is induced as ceramide is antagonised by GlcCer |
| **Repair** | **BRCA-1** | BRCA-1 is a tumor suppressor gene, which directly binds to phosphorylated CtIP protein and localizes CtIP to DNA double-strand breaks (DSBs), which facilitates DNA double-strand break repair. |
|  | **BRCA-2** | BRCA-2 is a tumor suppressor gene, which directly binds to RAD51 and facilitates loading of RAD51 on ssDNA. RAD51 forms a nucleoprotein filament on ssDNA and catalyses homologous recombination. |
|  | **p53** | p53 is a tumor suppressor gene, which responds to diverse cellular stresses to regulate target genes that induce cell cycle arrest, apoptosis, senescence, DNA repair, or changes in metabolism. |
| **Detoxification** | **Nrf-2** | NF-E2-related factor 2 (Nrf-2) was identified as the main transcription factor regulates the antioxidant response by introducing the expression of genes bearing an ARE in their regulatory regions, such as NQO1, GCS, and HO-1 |
|  | **Keap-1** | Kelch-like ECH-associated protein 1(Keap-1) interacts with Nrf-2 in a redox-sensitive manner and the dissociation of the proteins in the cytoplasm is followed by transportation of NF-E2-related factor 2 to the nucleus. |
|  | **γ-GCL** | Glutamate-cysteine ligase (GCL) is the ﬁrst and rate-limiting enzyme involved in the biosynthesis of glutathione, catalyzes the ligation of glutamate to cysteine. |
|  | **HO-1** | Heme oxygenase 1 (HO-1) is an inducible isoform in response to stress , through anti-oxidative, anti-apoptotic and anti-inflammatory actions |
|  | **HIF-1-α** | Hypoxia-inducible factor-1 (HIF-1), which is a heterodimer composed of an alpha and a beta subunit. HIF-1 functions as a master regulator of cellular and systemic homeostatic response to hypoxia |
|  | **GR** | Glutathione reductase(GR), is an enzyme that reduces glutathione disulfide (GSSG) to the sulfhydryl form GSH, which is an important cellular antioxidant. |
|  | **GST-π** | Glutathione S-transferase pi (GST pi) is a subgroup of GST family, which provides cellular protection against free radical and carcinogenic compounds due to its detoxifying function |
| **PKC-α** | | Protein kinase C (PKC) is a family of serine- and threonine-specific protein kinases that can reverse apoptosis through the scavenging of ROS as well as inhibition of PARP cleavage. |
| **EMT** | **E-cadherin** | E-cadherin, a cell-cell adhesion molecule, connects to the cytoskeleton through cytoplasmic catenins, and affects the adhesive, motile and morphological properties of cells. The loss of E-cadherin function contributes to increased invasion and metastasis in carcinoma cells. |
|  | **N-cadherin** | N-cadherin, a mesenchymal cadherin associated with the EMT, is crucial in cancer progression, with respect to both metastasis and to chemotherapy resistance. |
|  | **Snail** | Snail belongs to a family of zinc finger-containing transcriptional repressors, and triggers EMT associated with the acquisition of invasive and tumorigenic properties. |
|  | **ZEB-1** | ZEB-1, an E-box transcription repressor, is predominantly responsible for loss of E-Cadherin associated with a poor prognosis and resistance to epidermal growth factor receptor inhibitors. |
|  | **ZEB-2** | ZEB-2, an E-box transcription repressor, is predominantly responsible for loss of E-Cadherin associated with a poor prognosis and resistance to epidermal growth factor receptor inhibitors. |
|  | **Slug** | Slug encodes a member of the Snail family of C2H2-type zinc finger transcription factors. The encoded protein acts as a transcriptional repressor that binds to E-box motifs and is also likely to repress E-cadherin transcription. |
|  | **Twist** | The basic helix-loop-helix transcription factor Twist1 is a major regulator of mesenchymal phenotypes. It has also been recently identified as capable of mediating carcinoma metastasis and regulating tumor resistance. |
|  | **VIM** | Vimentin (VIM) is a type III intermediate filament protein, and it is a marker of epithelial-mesenchymal transition (EMT). The term EMT refers to mechanisms by which epithelial cells lose their differentiated characteristics and acquire mesenchymal feature. |
